# Effects of exenatide versus insulin glargine on body composition in overweight and obese T2DM patients: a randomized controlled trial

**DOI:** 10.1186/s12986-018-0295-6

**Published:** 2018-10-01

**Authors:** Ting-Ting Yin, Yan Bi, Ping Li, Shan-Mei Shen, Wei-Min Wang, Can Jiang, Cai-Xia Gao, Yan Wang, Li-Jun Gao, Da-Long Zhu, Wen-Huan Feng

**Affiliations:** 1 Department of Endocrinology, Drum Tower Hospital Affiliated to Nanjing University Medical School, Nanjing, China; 2 Department of Endocrinology, Drum Tower Clinical Hospital, Medical School of Southeast University, Zhongshan Road 321, Nanjing, 210008 China; 3 Department of Endocrinology, Jining No1. People’s Hospital, Shandong, China; 4 Department of Traditional Chinese Medicine, Yan’an People’s Hospital, Yan’an, China

**Keywords:** Body composition, Exenatide, Insulin glargine, Type 2 diabetes

## Abstract

**Background:**

Weight loss, especially fat mass reduction, helps to improve blood glucose control, insulin sensitivity, and β-cell function. This study aimed to compare the effect of exenatide and glargine on body composition in overweight and obese patients with type 2 diabetes (T2DM) who do not achieve adequate glycemic control with metformin.

**Methods:**

We performed a prospective, randomized study of 37 overweight or obese patients with T2DM who had inadequate glycemic control with metformin. The patients were treated with either exenatide or glargine for 16 weeks. Dual-energy X-ray absorptiometry was used to assess body composition.

**Results:**

Post-intervention weight, body mass index (BMI), waist circumference, body mass, and fat mass were lower in patients treated with exenatide, while weight and BMI significantly increased with glargine. Reductions in weight, BMI, body fat mass, and percent fat mass (except for gynoid) were greater with exenatide than with glargine, and percent lean tissue (other than the limbs) increased with exenatide. In all body regions except for the limbs, fat mass decreased with exenatide to a greater extent than lean tissue. Glucose control, insulin resistance, and β-cell function were not different between the treatment groups.

**Conclusions:**

For overweight and obese patients whose T2DM was inadequately controlled with metformin, exenatide and glargine achieved similar improvements in glycemic control, insulin sensitivity, and β-cell function.However, exenatide produced better weight and fat mass reduction, which were beneficial for blood glucose control. Our findings may guide the selection of appropriate drugs for glycemic and weight control.

**Trial registration:**

NCT02325960, registered 25 December 2014.

## Background

The prevalence of overweight and obesity has markedly increased worldwide [1, 2]. Obesity, particularly “android obesity” that is characterized by the accumulation of android-visceral fat, is considered a major risk factor for the development of insulin resistance [3] and type 2 diabetes (T2DM) [4, 5], and represents one of the most critical public health challenges [1, 6]. Increased fat mass associated with weight gain, especially abdominal weight gain during T2DM treatment, ultimately impedes the control of glycemia and other metabolic disorders [7].

Management of T2DM includes lifestyle modifications [8, 9] and use of antihyperglycemic drugs [10]. Oral medications that produce weight loss, especially metformin [10–12], are the mainstay of first-line treatments for T2DM. However, metformin monotherapy does not typically achieve good blood glucose control over an extended period. Glucagon-like peptide-1 (GLP-1) receptor agonists [10, 13–16] and basal insulin [14, 15, 17] are both choices as pharmaceutical approaches to glycemic treatment for T2DM patients who use metformin alone but nevertheless have poorly controlled glycemia. However, previous studies found that these two types of drugs had different effects on body weight and android obesity [18–22]. Furthermore, GLP-1 receptor agonists exhibit potent blood glucose reduction only during hyperglycemia, during which they increase insulin secretion and reduce glucagon secretion in a glucose-dependent manner [23, 24]. Clinical studies have demonstrated that GLP-1 receptor agonists reduce body weight and visceral and hepatic fat deposits, and also improve hepatic insulin sensitivity in obese patients with T2DM [18, 25–27]. Moreover, exenatide, a GLP-1 receptor antagonist, is associated with a statistically significant reduction in total fat mass, mainly in the trunk [19], whereas the basal insulin glargine reduces liver fat content and improves hepatic insulin sensitivity [28]. Glargine is also associated with significantly increased total, trunk, and limb fat mass as well as limb lean tissue [21, 22].

It is not clear whether GLP-1 receptor agonists or basal insulin can achieve better control of body composition in overweight or obese T2DM patients who have inadequate glycemic control with metformin. There are no reports on the effects of exenatide or glargine on trunk and android body composition, which might be more associated with long-term glycemic control in patients with T2DM following metformin treatment. To fill this knowledge gap, we used dual-energy X-ray absorptiometry (DXA) to compare changes in body composition, including fat mass and lean tissue, after using exenatide or glargine in combination with metformin in overweight and obese T2DM patients with poor glycemic control. Our study aims to assist in selecting optimal therapies for glucose reduction and weight control, especially in overweight and obese patients with T2DM.

## Materials and methods

### Subjects

Forty-five overweight and obese T2DM patients with poor glycemic control despite metformin monotherapy were randomized using arbitrary computer-generated numbers to receive exenatide or glargine treatments for 16 weeks in addition to their current metformin treatment. Block randomization was performed by third-party statisticians. The inclusion criteria were as follows: 1) patients with T2DM receiving a stable metformin dose of ≥1.5 g/d for > 8 weeks; 2) age between 18 and 70 years; 3) glycated hemoglobin (HbA1c) between 7.0 and 10.0%; and 4) body mass index (BMI) ≥24 kg/m^2^ (based on the China Obesity Task Force criteria) [29]. Exclusion criteria included: 1) allergy to related drugs; 2) impaired renal function (serum creatinine ≥1.5 mg/dL or ≥ 133 μmol/L); 3) diseases causing acute or chronic hypoxia such as respiratory failure or stroke; 4) liver dysfunction (alanine aminotransferase [ALT] and aspartate aminotransferase [AST] ≥3 times higher than the high normal limit) or acute alcoholism; 5) history of cardiovascular disease in the past 12 months; 6) proliferative retinopathy; 7) positive pregnancy test result, breastfeeding mother, or not willing to use the appropriate contraceptive methods; 8) systemic corticosteroid therapy used in the past two months; 9) type 1 diabetes; or 10) use of other experimental drugs during the preceding 30 days. The study protocol was approved by the Research Ethics Board of Drum Tower Hospital affiliated with Nanjing University Medical School (Protocol: AF/SQ-2014-072-01). Adverse events (AEs) were collected and recorded in case report form, and serious adverse events (SAEs) were reported in written form to the Institutional Review Board of the Drug Clinical Trial Agency Office and the Research Ethics Board of Drum Tower Hospital, affiliated to Nanjing University Medical School.

### Study protocol

**Fig. 1:**
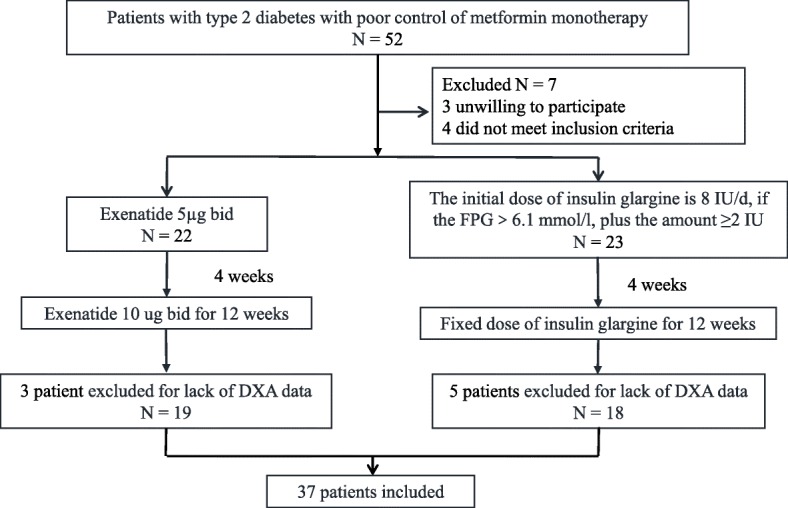
Flow chart of the study participants

This prospective, randomized, and parallel design trial lasted 16 weeks. For participants assigned to receive exenatide, the initial dosage was 5 μg twice daily for 4 weeks followed by 10 μg twice daily for the remainder of the trial. For basal insulin glargine, the starting dose was 8 IU once daily, followed by a titrated dosage of ≥2 IU every 3 days based on fasting blood glucose (FBG) levels until the peripheral blood glucose level reached 6.1 mmol/L, following which a maintenance dose with fixed glargine was administered for the remaining 12 weeks (Fig. 1). All patients included in the study provided written informed consent. Information regarding proper diet and exercise was provided to all patients in the two groups. Patients enrolled in the study were provided free antidiabetic drugs and related laboratory examinations during follow-up. Data from the same trial were published in a previous study [30] that compared the effects of exenatide and insulin glargine on glycemic variability using a CGMS® (Glod, Medtronic); in that study, exenatide was found to be more effective in reducing body weight and BMI although both showed similar efficacy in achieving glycemic control.

### Measurements

Fat mass and lean tissue in the total body, trunk, android regions, gynoid regions, and limbs were measured using DXA (Lunar iDXA; Encore 13.4) at baseline and at the end of the study. Body weight, waist circumference (WC), and blood pressure were measured at baseline and every four weeks.

All participants were administered an identical 85 g carbohydrate-equivalent meal at baseline and at the end of the treatment period. Plasma glucose and insulin levels were measured at 0, 30, 60, and 120 min after the meal. Fasting serum HbA1c, ALT, AST, total cholesterol (TC), triglyceride, high-density lipoprotein cholesterol, low-density lipoprotein cholesterol (LDL-C), uric acid, and creatinine levels were measured at baseline and after 16 weeks of intervention.

### Statistical analyses

The study was non-blinded, and all analyses were completed by an independent statistician using SPSS version 18.0 software (SPSS Inc., Chicago, IL, USA). The a priori power calculations for sample size were previously described [30]. A paired Student’s t-test was used when comparing pre- and post-intervention changes within each intervention group. Analysis of covariance was used to test for differences between the treatment groups after adjusting for baseline values. Effects of changes in body composition in relation to FBG and HbA1c were identified using Pearson’s correlation analysis and multivariate logistic regression. The non-inferiority margin of 0.30% in HbA1c was selected based on previous guidance [31], and was considered clinically significant if the upper limit of the 95% confidence interval (CI) of the treatment difference was less than or equal to 0.3% points [32]. The data are expressed as means ± standard errors (SE). Statistical significance was defined as *P <* 0.05.

## Results

### Baseline values

Thirty-seven of the 45 randomized patients completed the study; three patients in the exenatide group and five in glargine were excluded for lack of DXA data (Fig. 1). Baseline clinical characteristics of the study population were similar between groups (Table 1). Nineteen subjects (12 men and seven women) aged 47.6 ± 2.5 years with a mean T2DM duration of 7.0 ± 1.2 years were treated with exenatide. In the glargine group, 18 participants (12 men and six women) aged 48.3 ± 2.3 years and T2DM duration of 4.4 ± 0.7 years completed treatment.

**Table 1:** Subject characteristics, insulin sensitivity, and β-cell function at baseline and after treatment

	Exenatide			Insulin glargine			Estimated treatment difference, Exenatide vs Insulin glargine Mean (95% CI)	*P* Decreased value between two groups
	Pre Mean + SE	Post Mean ± SE	*P*	Pre Mean ± SE	Post Mean ± SE	*P*		
Number (n)	19	–	–	18	–	–	–	–
Sex (male/female)	12/7	–	–	12/6	–	–	–	–
Age	47.6 ± 2.5	–	–	48.3 ± 2.3	–	–	–	–
Diabetes duration	7.0 ± 1.2	–	–	4.4 ± 0.7	–	–	–	–
Weight (kg)	80.8 ± 2.4	77.3 ± 2.4	< 0.001	75.1 ± 1.8	76.0 ± 1.8	0.048	− 4.5 (− 6.3 to − 2.7)	< 0.001
BMI (kg/m^**2**^)	28.1 ± 0.5	26.9 ± 0.5	< 0.001	27.0 ± 0.6	27.3 ± 0.5	0.048	−1.6 (− 2.2 to − 0.9)	< 0.001
Waist circumference (cm)	96.5 ± 1.5	93.5 ± 1.6	0.012	93.9 ± 1.7	93.4 ± 1.7	0.361	−2.6 (− 5.0 to − 0.1)	0.070
SBP (mmHg)	119.4 ± 3.5	120.9 ± 3.2	0.600	114.7 ± 2.9	118.3 ± 3.9	0.329	−2.1 (− 11.3 to 7.0)	0.943
DBP (mmHg)	77.7 ± 1.9	79.8 ± 1.5	0.293	77.2 ± 2.0	74.2 ± 2.2	0.205	5.2 (−1.0 to 11.3)	0.034
ALT (U/L)	31.8 ± 4.3	31.0 ± 4.1	0.825	36.8 ± 4.8	29.5 ± 3.8	0.035	6.5 (− 3.3 to 16.3)	0.290
AST (U/L)	21.9 ± 2.2	21.4 ± 2.2	0.766	24.6 ± 2.1	22.6 ± 2.0	0.191	1.4 (− 3.1 to 5.9)	0.756
TG (mmol/L)	1.8 ± 0.2	1.6 ± 0.2	0.243	1.7 ± 0.2	1.5 ± 0.2	0.301	0.0 (− 0.5 to 0.5)	0.778
TC (mmol/L)	4.5 ± 0.3	4.1 ± 0.3	0.020	4.3 ± 0.2	4.3 ± 0.2	0.979	− 0.5 (− 1.0 to 0.1)	0.113
HDL-C (mmol/L)	1.0 ± 0.1	1.0 ± 0.1	0.176	1.0 ± 0.1	1.1 ± 0.1	0.107	− 0.2 (− 0.3 to 0.0)	0.040
LDL-C (mmol/L)	2.4 ± 0.2	2.1 ± 0.2	0.009	2.3 ± 0.1	2.3 ± 0.1	0.914	− 0.3 (− 0.7 to 0.0)	0.091
FBG (mmol/L)	8.7 ± 0.5	6.8 ± 0.4	0.002	9.0 ± 0.5	6.9 ± 0.4	< 0.001	0.2 (− 1.2 to 1.6)	0.978
30-min glucose (mmol/L)	10.8 ± 0.7	9.1 ± 0.9	0.070	12.1 ± 0.7	9.9 ± 0.6	0.001	0.5 (− 1.6 to 2.7)	0.867
60-min glucose (mmol/L)	14.1 ± 1.0	11.7 ± 1.1	0.087	14.8 ± 0.7	11.9 ± 0.5	< 0.001	0.5 (− 2.4 to 3.4)	0.976
120-min glucose (mmol/L)	13.3 ± 0.9	11.7 ± 0.8	0.101	14.0 ± 1.0	12.3 ± 0.9	0.123	0.0 (− 2.8 to 2.8)	0.691
Fasting insulin (uIU/mL)	10.1 ± 1.3	10.1 ± 1.7	0.984	11.0 ± 1.0	10.0 ± 1.9	0.616	1.1 (−4.3 to 6.5)	0.822
30 min insulin (uIU/mL)	19.5 ± 3.0	17.8 ± 3.9	0.593	19.2 ± 2.2	23.8 ± 5.0	0.345	−6.3 (− 17.6 to 5.0)	0.272
60 min insulin (uIU/mL)	28.7 ± 5.1	33.8 ± 8.8	0.489	33.2 ± 3.7	36.2 ± 6.4	0.660	2.0 (− 18.2 to 22.2)	0.906
120 min insulin (uIU/mL)	34.8 ± 5.5	39.5 ± 8.8	0.414	40.6 ± 6.3	48.9 ± 8.9	0.357	− 3.6 (− 24.2 to 17.1)	0.692
1 / HOMA-IR (μIU/mL. mmol/L)	0.4 ± 0.1	0.5 ± 0.1	0.035	0.3 ± 0.0	0.5 ± 0.1	0.007	0.0 (− 0.3 to 0.2)	0.886
ISI_M_ (μIU/mL. mmol/L)	4.5 ± 0.7	7.1 ± 1.1	0.034	3.7 ± 0.6	5.3 ± 0.8	0.040	0.9 (− 1.9 to 3.8)	0.292
HOMA-β (IU/mol)	50.4 ± 9.9	124.4 ± 48.2	0.098	54.6 ± 10.9	87.5 ± 30.5	0.315	41.0 (− 68.8 to 150.8)	0.418
InsAUC30 / GluAUC30 (IU/mol)	11.9 ± 1.9	15.5 ± 3.3	0.106	11.3 ± 1.6	15.0 ± 3.2	0.225	0.0 (− 7.3 to 7.3)	0.977
InsAUC120 / GluAUC120 (IU/mol)	17.3 ± 3.2	22.3 ± 5.8	0.118	16.7 ± 2.9	22.6 ± 5.0	0.232	− 0.9 (− 11.9 to 10.1)	0.839
DI 30 (mmol/L. mmol/L)	42.5 ± 5.2	72.2 ± 12.4	0.012	35.0 ± 4.5	61.0 ± 6.7	< 0.001	3.8 (− 21.0 to 28.5)	0.869
DI 120 (mmol/L. mmol/L)	62.2 ± 9.5	100.6 ± 18.0	0.019	50.8 ± 7.1	90.2 ± 10.8	0.004	−1.0 (− 39.4 to 37.4)	0.992
HBA1c (%)	8.0 ± 0.2	6.8 ± 0.3	< 0.001	8.2 ± 0.2	7.1 ± 0.2	0.001	− 0.1 (− 0.7 to 0.6)	0.469

*BMI* body mass index, *SBP* systolic blood pressure, *DBP* diastolic blood pressure, *ALT* alanine aminotransferase, *AST* aspartate aminotransferase, *TG* triglyceride, *TC* total cholesterol, *LDL-C* low-density lipoprotein cholesterol, *HDL-C* high-density lipoprotein cholesterol, *FBG* fasting blood glucose, *1/HOMA-IR* 1/homeostasis model assessment of insulin resistance, *ISI*_*M*_ Matsuda insulin sensitivity index, *HOMA-β* basal homeostasis model assessment of insulin secretion, InsAUC30/GluAUC30 was calculated as the total insulin area under the curve divided by the total glucose area under the curve during the first 30 min of the 75-g oral glucose tolerance test (OGTT); InsAUC120/GluAUC120 was calculated as the total insulin area under the curve divided by the total glucose area under the curve during the 120 min of the OGTT; DI30: disposition index 30; DI120: disposition index 120; HbA1c: glycated hemoglobin. *P*-values < 0.05 were considered indicative of statistically significant differences between the groups

### Body weight, BMI, and WC

Body weight and BMI (both *P* < 0.001) significantly decreased after exenatide intervention, while body weight and BMI (both *P* = 0.048) significantly increased after glargine intervention (Table 1). After treatment, WC significantly decreased (*P* = 0.012) in the exenatide group but did not change in the glargine group. The exenatide treatment group had greater decreases in body weight (*△* = − 4.5 kg; 95% CI, − 6.3 to − 2.7 kg) and BMI (*△* = − 1.6 kg/m^2^; 95%CI, − 2.2 to − 0.9 kg/m^2^) than the insulin glargine group (*P* < 0.001) (Table 1).

### Body fat mass and lean tissue

A significant reduction of body fat mass was achieved with exenatide treatment, including total (*△* = − 2.1 kg; 95% CI, − 3.0 to − 1.2 kg; *P* < 0.001), trunk (*△* = − 1.3 kg; 95% CI, − 2.0 to − 0.7 kg; *P* < 0.001), limb (*△* = − 0.7 kg; 95% CI, − 1.1 to − 0.4 kg; *P* < 0.001), android (*△* = − 0.3 kg; 95% CI, − 0.5 to − 0.1 kg; *P* = 0.001), and gynoid (*△* = − 0.2 kg; 95% CI, − 0.3 to − 0.1 kg; *P* = 0.002). However, no changes were observed after insulin glargine intervention (Table 2). There was a significantly greater decrease in fat mass in the exenatide group (including total, trunk, limb, android, and gynoid fat) (Table 2).

**Table 2:** Fat mass and lean tissue distribution at baseline and after treatment

	Exenatide			Insulin glargine			Estimated treatment difference, Exenatide vs Insulin glargine Mean (95% CI)	*P* Decreased value between two groups
	Pre Mean ± SE	Post Mean ± SE	*P*	Pre Mean ± SE	Post Mean ± SE	*P*		
Total fat mass (kg)	25.8 ± 1.0	23.7 ± 1.0	< 0.001	23.0 ± 1.1	23.0 ± 1.3	0.989	− 2.1 (− 3.2 to − 1.0)	0.001
Trunk fat (kg)	16.0 ± 0.6	14.7 ± 0.6	< 0.001	14.4 ± 0.9	14.4 ± 1.0	0.847	− 1.3 (− 2.0 to − 0.5)	0.001
Limb fat (kg)	8.6 ± 0.4	7.9 ± 0.4	< 0.001	7.5 ± 0.4	7.5 ± 0.4	0.825	− 0.8 (− 1.2 to 0.3)	0.005
Android fat (kg)	2.8 ± 0.1	2.5 ± 0.1	0.001	2.5 ± 0.2	2.4 ± 0.2	0.153	− 0.2 (− 0.4 to 0.1)	0.019
Gynoid fat (kg)	3.2 ± 0.2	3.0 ± 0.2	0.002	2.8 ± 0.2	2.8 ± 0.2	0.933	− 0.2 (− 0.3 to − 0.1)	0.018
Total fat mass (%)	32.4 ± 1.4	30.7 ± 1.4	< 0.001	31.0 ± 1.3	30.9 ± 1.5	0.619	− 1.5 (− 2.5 to − 0.5)	0.005
Trunk fat (%)	38.9 ± 1.4	36.5 ± 1.5	< 0.001	37.3 ± 1.6	36.9 ± 1.8	0.465	− 2.0 (− 3.5 to − 0.6)	0.006
Limb fat (%)	26.1 ± 1.5	25.0 ± 1.5	0.001	24.8 ± 1.3	24.8 ± 1.4	0.908	− 1.1 (− 1.9 to − 0.3)	0.010
Android fat (%)	42.0 ± 1.4	39.1 ± 1.5	< 0.001	40.5 ± 1.7	39.8 ± 1.0	0.278	− 2.2 (− 4.0 to − 0.4)	0.018
Gynoid fat (%)	28.4 ± 1.7	27.6 ± 1.6	0.046	26.5 ± 1.3	26.5 ± 1.4	0.973	− 0.8 (− 1.9 to 0.2)	0.128
Total lean tissue (kg)	52.0 ± 2.1	51.6 ± 2.2	0.278	48.4 ± 1.3	48.7 ± 1.4	0.445	− 0.7 (− 1.7 to 0.4)	0.216
Trunk lean tissue (kg)	24.6 ± 1.0	24.9 ± 1.0	0.123	23.2 ± 0.6	23.3 ± 0.6	0.600	0.2 (− 0.4 to 0.8)	0.460
Limb lean tissue (kg)	23.7 ± 1.1	23.0 ± 1.1	0.011	21.7 ± 0.8	21.8 ± 0.8	0.514	− 0.8 (− 1.4 to − 0.2)	0.019
Android lean tissue (kg)	3.8 ± 0.2	3.8 ± 0.2	0.750	3.5 ± 0.1	3.5 ± 0.1	0.863	0.0 (− 0.2 to 0.1)	0.013
Gynoid lean tissue (kg)	8.0 ± 0.3	7.9 ± 0.4	0.066	7.5 ± 0.2	7.5 ± 0.2	0.731	− 0.2 (− 0.4 to 0.0)	0.110
Total lean tissue (%)	64.3 ± 1.3	65.8 ± 1.3	< 0.001	65.5 ± 1.3	65.6 ± 1.5	0.693	1.4 (0.4 to 2.4)	0.007
Trunk lean tissue (%)	59.1 ± 1.3	61.4 ± 1.4	0.001	60.7 ± 1.6	61.0 ± 1.8	0.540	2.0 (0.5 to 3.5)	0.007
Limb lean tissue (%)	69.9 ± 1.5	70.8 ± 1.5	0.007	70.9 ± 1.2	71.0 ± 1.3	0.895	0.8 (0.0 to 1.7)	0.050
Android lean tissue (%)	57.2 ± 1.4	60.1 ± 1.5	< 0.001	58.7 ± 1.7	59.3 ± 2.0	0.323	2.2 (0.4 to 4.1)	0.017
Gynoid lean tissue (%)	69.1 ± 1.6	70.0 ± 1.6	0.049	70.8 ± 1.3	70.8 ± 1.4	0.994	0.8 (0.0 to 1.6)	0.019

The percent fat mass was calculated by dividing local fat mass by weight of the same body regions; the percent lean tissue was calculated by dividing local lean mass by weight of the same body regions. *P*-values < 0.05 were considered indicative of statistically significant differences between the groups

Percent total fat mass (*△* = − 1.7%; 95% CI, − 2.4 to − 0.9%; *P* < 0.001), trunk fat (*△* = − 2.4%; 95% CI, − 3.5 to − 1.2%; *P* < 0.001), limb fat (*△* = − 1.1%; 95% CI, − 1.7 to − 0.5%; *P* = 0.001), android fat (*△* = − 2.9%; 95% CI, − 4.2 to − 1.5%; *P* < 0.001), and gynoid fat (*△* = − 0.9%; 95% CI, − 1.7 to 0.0%; *P* = 0.046) decreased with exenatide, but were unchanged with glargine (Table 2). Exenatide was superior to glargine in reducing the percentage of fat (total fat mass: *P* = 0.005; trunk fat: *P* = 0.006; limb fat: *P* = 0.010; android fat: *P* = 0.018) (Table 2).

After treatment with exenatide, limb lean tissue significantly decreased (*△* = − 0.7 ± 0.2 kg, *P =* 0.011); this decreased was greater with exenatide than with glargine (*P* = 0.019; Table 2).

**Fig. 2:**
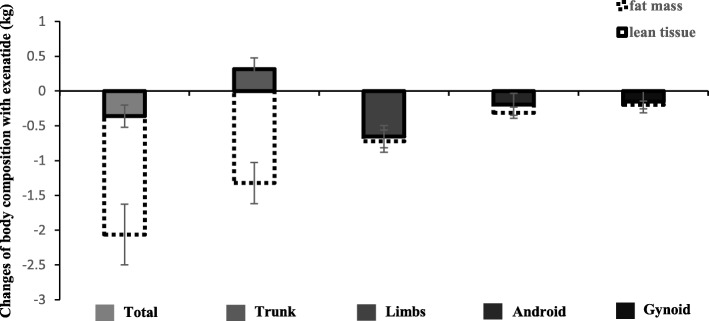
Changes in fat mass and lean tissue in the total body, trunk, limbs, and android and gynoid regions following exenatide treatment

Exenatide significantly increased the percentage of total body lean tissue (*△* = 1.5%; 95% CI, 0.8 to 2.3%; *P* < 0.001), trunk (*△* = 2.3%; 95% CI, 1.1 to 3.5%; *P* = 0.001), limbs (*△* = 0.9%; 95% CI, 0.3 to 1.5%; *P* = 0.007), android regions (*△* = 2.9%; 95% CI, 1.5 to 4.3%; *P* < 0.001), and gynoid regions (*△* = 0.9%; 95% CI, 0.0 to 1.7%; *P* = 0.049), while they were unchanged with glargine (Table 2). There appeared to be greater increases in the percentage of lean tissue in the total body (1.4%; 95% CI, 0.4 to 2.4%; *P* = 0.007), trunk (2.0%; 95% CI, 0.5 to 3.5%; *P* = 0.007), android regions (2.2%; 95% CI, 0.4 to 4.1%; *P* = 0.017), and gynoid regions (0.8%; 95% CI, 0.0 to 1.6%; *P* = 0.019) (Table 2) in the exenatide group than in the insulin glargine group. Generally, exenatide treatment reduced fat mass more than it reduced lean tissue (Fig. 2).

### Correlative analysis and multiple regression

In the exenatide group, there were significant positive correlations between *△*fat mass (including total, trunk, limb, and android fat [0 < *r* < 1, *P <* 0.05] and each of *△*FBG and *△*HbA1c. The *△*fat mass percentage, including total, trunk, and android fat, was positively correlated with *△*FBG and *△*HbA1c (0 < *r* < 1, *P <* 0.05). Furthermore, there was an inverse correlation between*△*lean tissue percentage (comprising total, trunk, and android lean tissue) and each of *△*FBG and *△*HbA1c (− 1 < *r* < 0, *P <* 0.05; Table 3).

**Table 3:** Correlations of body composition measurements with FBG and HbA1c following exenatide treatment

	Changes in FBG (mmol/L)		Changes in HbA1c (%)	
	*r*	*p*	*r*	*p*
Changes in total fat (kg)	0.629	0.005	0.644	0.004
Changes in trunk fat (kg)	0.611	0.007	0.628	0.005
Changes in limbs fat (kg)	0.564	0.015	0.578	0.012
Changes in Android fat (kg)	0.621	0.006	0.772	<0.001
Changes in total fat (%)	0.519	0.018	0.631	0.005
Changes in trunk fat (%)	0.549	0.018	0.640	0.004
Changes in Android fat (%)	0.540	0.021	0.718	0.001
Changes in total lean (%)	−0.513	0.030	− 0.645	0.004
Changes in trunk lean (%)	−0.536	0.022	−0.639	0.004
Changes in Android lean (%)	−0.528	0.024	−0.689	0.002

*FBG* fasting blood glucose, *HbA1c* glycated hemoglobin

In the exenatide group, multiple regression analysis was used to evaluate variables independently associated with *△*FBG and *△*HbA1c. We found that *△*total fat mass and percentage *△*trunk fat mass were correlated with *△*FBG (*R*^2^ = 0.397, *P* = 0.002; and *R*^2^ = 0.298, *P* = 0.009, respectively) and that *△*android fat mass in terms of both mass and percent mass were correlated with *△*HbA1c (*R*^2^ = 0.547, *P* < 0.001; *R2* = 0.454, *P* = 0.001, respectively).

### Glycemic control, insulin sensitivity, and β-cell function

Exenatide and glargine significantly reduced FBG (exenatide, *△* = − 2.0 mmol/L, *P* = 0.002; glargine, *△* = − 2.1 mmol/L, *P* < 0.001) and HbA1c values (exenatide, *△* = − 1.20%, *P* < 0.001; glargine, *△* = − 1.1%, *P* = 0.001). The reduction in FBG and HbA1c levels did not differ between the two groups. Blood glucose values at 30 min (*△* = − 2.2 mmol/L, *P* = 0.001) and 60 min (*△* = − 2.9 mmol/L, *P* < 0.001) also decreased with glargine treatment (Table 1).

The mean decrease in HbA1c from baseline was similar between treatments. The statistical results showed that the upper limit of the 95% CI was 0.74, which was greater than the previously set value. Thus, it was not yet possible to argue that exenatide was less efficient than insulin glargine in reducing HbA1c concentrations.

Exenatide and glargine increased the 1/homeostasis model assessment of insulin resistance (1/HOMA-IR) (exenatide from 0.4 ± 0.1 to 0.5 ± 0.1 μIU/mL. mmol/L, *P* = 0.035; glargine from 0.3 ± 0.0 to 0.5 ± 0.1 μIU/mL. mmol/L, *P* = 0.007), the Matsuda insulin sensitivity index (ISI_M_) (exenatide from 4.5 ± 0.7 to 7.1 ± 1.1 μIU/mL. mmol/L, *P* = 0.034, glargine from 3.7 ± 0.6 to 5.3 ± 0.8 μIU/mL. mmol/L, *P* = 0.040), disposition index 30 (exenatide from 42.5 ± 5.2 to 72.2 ± 12.4 mmol/L. mmol/L, *P* = 0.012; glargine from 35.0 ± 4.5 to 61.0 ± 6.7 mmol/L. mmol/L, *P* < 0.001), and disposition index 120 (exenatide from 62.2 ± 9.5 to 100.6 ± 18.0 mmol/L. mmol/L, *P* = 0.019; glargine from 50.8 ± 7.1 to 90.2 ± 10.8 mmol/L. mmol/L, *P* = 0.004). However, there were no statistical differences between the two groups. Moreover, the basal homeostasis model assessment of insulin secretion (HOMA-β), the early-phase ratio of the area under the curve of insulin to glucose during 0–30 min (InsAUC30/GluAUC30), and total-phase InsAUC120/GluAUC120 did not differ between the pre-treatment and post-treatment values in the two groups (Table 1).

### Lipid profiles and liver function

After 16 weeks of treatments, TC (*△* = − 0.5 mmol/L, *P* = 0.020) and LDL-C (*△* = − 0.3 mmol/L, *P* = 0.009) significantly decreased in the exenatide group but were unchanged in the glargine group. Conversely, ALT (*△* = − 7.3 IU/L, *P* = 0.035) significantly decreased with glargine, but did not change with exenatide. The extent of reduction in TC, LDL-C, and ALT did not differ between the two groups (*P >* 0.05; Table 1).

### AEs

In the exenatide group, 17 subjects had gastrointestinal intolerance/appetite suppression, three subjects had nausea that resolved over time, and four subjects had abdominal distension. No subjects in the insulin glargine-treated group experienced gastrointestinal intolerance or any other AEs. Moreover, no hypoglycemia-related events occurred in these patients, which might due to the absence of preprandial insulin and insulin-stimulating drugs. No participants withdrew from the study due to these AEs, and no SAEs occurred whatsoever.

## Discussion

A notable finding in our study was that exenatide was superior to glargine in reducing absolute and percent fat mass and in increasing the percent lean tissue in different parts of the body, while improvements in FBG, HbA1c, insulin sensitivity, and β-cell function between the two groups were similar after 16 weeks of treatment. Weight loss by exenatide was mainly due to decreased body fat content rather than decreased lean tissue.

The LEAD-2 trial showed that the percentage of total fat mass decreased significantly more after 26 weeks of treatment with the GLP-1 receptor agonist liraglutide at doses of 1.2 and 1.8 mg combined with metformin than after treatment with glimepiride/metformin [18]. The total lean body tissue measured by DXA or computed tomography decreased in the 0.6, 1.2, and 1.8 mg metformin groups, and reductions in visceral adipose tissue were greater than those in abdominal subcutaneous adipose tissue in the 1.2 and 1.8 mg metformin groups. With respect to GLP-1 agonist monotherapy, the LEAD-3 trial showed that the total fat mass and percent total fat were significantly decreased with liraglutide but increased with glimepiride [18]. Consistent with previous findings, we found that fat mass reduction by exenatide/metformin played a greater role in weight loss than did lean tissue reduction. Weight gain, especially by fat accumulation, impedes long-term glycemic control; hence, this combination may be more beneficial to reducing excess body fat in overweight and obese T2DM and assist in the long-term regulation of blood glucose [7]. Similar to the current 16-week study, a 1-year randomized study combining exenatide or glargine with metformin in 69 patients with T2DM showed that, in contrast to glargine, exenatide significantly reduced total fat mass and trunk fat mass. However, in contrast to the current study, no differences in total lean tissue were found [19]. Furthermore, we found that exenatide decreases fat mass in the total body, trunk, limbs, android regions, and gynoid regions to a greater extent than glargine. Our study also revealed that exenatide decreased the percentage of fat in the total body, trunk, android regions, and limbs, and that this was accompanied by an increase in the percentage of lean tissue in the total body, trunk, android regions, and gynoid regions. Fat mass in the trunk, especially in the android region, has been shown to exhibit a stronger direct correlation with cardiovascular disease risk factors than has fat mass in other parts of the body [4, 5, 33–37]. In the current study, the reduced fat mass and increased lean tissue observed following exenatide treatment, especially in the trunk and android regions, was also related to improvements in glycemic control. These data suggest that exenatide may help reduce the risk of cardiovascular disease in patients with T2DM.

In a study by Bi et al., the effects of exenatide and premixed insulin on body fat distributions were compared using magnetic resonance imaging (MRI); they showed that exenatide markedly reduced both visceral and subcutaneous fat while premixed insulin did not [20]. In contrast to their study, DXA (as used in our study) might be better for measuring drug-induced changes in body fat composition than MRI; distinguishing and measuring body fat in the trunk, android, and gynoid regions; and evaluating the effect of exenatide and basal insulin on fat mass and lean tissue in different parts of the human body.

We found that glargine treatment produced weight gain with little change in fat mass and lean tissue. In contrast, a 6-month study comparing glargine to detemir in insulin-naïve patients with T2DM who had inadequate disease control found that the former significantly increased body weight, total fat mass, trunk fat mass, fat mass, and lean mass in limbs, while the latter significantly decreased truncal lean mass [21]. Unlike glargine, detemir regulates central appetite via more robust signal transduction in the hypothalamus and cerebral cortex than in the periphery; thus, glargine might be associated with more weight gain [38, 39].

Weight loss, especially adipose tissue reduction, typically improves blood glucose control in accordance with the degree of weight loss and also improves insulin sensitivity and β-cell function [40–42]. Nevertheless, we found similar improvements in insulin sensitivity as measured by 1/HOMA-IR and ISI_M_, as well as similar improvements in insulin release and β-cell function as determined via the HOMA-β, InsAUC30/GluAUC30, InsAUC120/GluAUC120, and disposition index in response to exenatide or glargine. This may be a function of early insulin sensitivity and β-cell function improvement as a consequence of glycemic control, while the effect of weight loss on insulin sensitivity and β-cell function may require a longer time. Weight loss, especially android fat mass reduction, is related to insulin resistance improvement [20, 43]; the positive relationship between fat mass reduction and the decreases in FBG and HbA1c only with exenatide in the current study suggests a more prolonged beneficial effect of exenatide on insulin sensitivity and β-cell function improvement.

Our study had some limitations. Only 37 patients participated, which may negatively influence the statistical power; moreover, the 16-week period may not have been sufficient to evaluate the additional benefits of weight and fat mass reduction on insulin sensitivity and β-cell function, as has previously been reported [20, 43]. A blinded multi-center research study with a larger number of patients is required to confirm our findings. Furthermore, in addition to DXA, body composition can also be measured using computed tomography [18] or MRI [20]; however, these methods are expensive, require a longer examination time, and expose the patients to greater amounts of radiation. The dosage of insulin glargine was titrated only in the first four weeks pursuant to our research design; hence, a fixed dosage of insulin glargine during the remaining 12 weeks may not be conducive to glucose control.

## Conclusions

Our results showed that exenatide and glargine produce similar improvements in glycemic control, insulin sensitivity, and β-cell function in overweight or obese patients with T2DM who are unable to achieve adequate glycemic control with metformin monotherapy. Our data showed that exenatide was superior to glargine in terms of reduced body weight, BMI, fat mass, and percent fat mass. Additionally, weight loss with exenatide was mainly a consequence of decreased fat mass rather than lean tissue mass, which may help improve glycemic and weight control.

## Abbreviations

1/HOMA-IR: The 1/homeostasis model assessment of insulin resistance
AEs: Adverse events
ALT: Alanine aminotransferase
AST: Aspartate aminotransferase
BMI: Body mass index
DXA: Dual-energy x-ray absorptiometry
FBG: Fasting blood glucose
GLP-1: Glucagon-like peptide-1
HbA1c: Glycated hemoglobin
HOMA-β: Basal homeostasis model assessment of insulin secretion
InsAUC*x*/GluAUC*x*: ratio of the area under the curve of insulin to glucose during 0–*x* min
ISI_M_: The Matsuda insulin sensitivity index
LDL-C: Low-density lipoprotein cholesterol
MRI: Magnetic resonance imaging
SAEs: Serious adverse events
T2DM: Type 2 diabetes
TC: Total cholesterol
WC: Waist circumference
